# Laryngomalacia: Disease Presentation, Spectrum, and Management

**DOI:** 10.1155/2012/753526

**Published:** 2012-02-27

**Authors:** April M. Landry, Dana M. Thompson

**Affiliations:** ^1^Department of Otolaryngology, Head and Neck Surgery, Mayo Clinic Arizona, Phoenix, AZ 85054, USA; ^2^Division of Pediatric Otolaryngology, Department of Otorhinolaryngology, Head and Neck Surgery, Mayo Clinic Children's Center and Mayo Eugenio Litta Children's Hospital, Mayo Clinic Rochester, 200 First Street SW, Gonda 12, Rochester, MN 55905, USA

## Abstract

Laryngomalacia is the most common cause of stridor in newborns, affecting 45–75% of all infants with congenital stridor. The spectrum of disease presentation, progression, and outcomes is varied. Identifying symptoms and patient factors that influence disease severity helps predict outcomes. *Findings*. Infants with stridor who do not have significant feeding-related symptoms can be managed expectantly without intervention. Infants with stridor and feeding-related symptoms benefit from acid suppression treatment. Those with additional symptoms of aspiration, failure to thrive, and consequences of airway obstruction and hypoxia require surgical intervention. The presence of an additional level of airway obstruction worsens symptoms and has a 4.5x risk of requiring surgical intervention, usually supraglottoplasty. The presence of medical comorbidities predicts worse symptoms. *Summary*. Most with laryngomalacia will have mild-to-moderate symptoms and not require surgical intervention. Those with gastroesophageal reflux and/or laryngopharyngeal reflux have symptom improvement from acid suppression therapy. Those with severe enough disease to require supraglottoplasty will have minimal complications and good outcomes if multiple medical comorbidities are not present. Identifying patient factors that influence disease severity is an important aspect of care provided to infants with laryngomalacia.

## 1. Introduction

Laryngomalacia is the most common cause of stridor in newborns, affecting 45–75% of all infants with congenital stridor [1]. The stridor can be overwhelming to parents and caregivers. The high-pitched noise of stridor is created by airflow through an area of obstruction. In laryngomalacia the supraglottic structures collapse into the airway during the inspiratory phase of respiration which produces inspiratory stridor. Most infants with laryngomalacia will have mild symptoms and a benign disease course that resolves by the age of 12 to 24 months; however, it is important to recognize that not all cases of laryngomalacia have a benign course [1]. Once the condition is diagnosed and differentiated from other causes of stridor, most mild cases can be followed expectantly by their pediatrician and referred back to an otolaryngology if symptoms worsen. The purpose of this paper is to review the disease presentation spectrum, highlighting symptoms and patient factors that predict which infants may worsen and require intervention or comanagement with an otolaryngologist. Supraglottoplasty is the mainstay surgical management. Tracheotomy to bypass the obstruction is rarely performed and reserved for surgical failures or children with multiple medical comorbidities.

## 2. Presentation

Laryngomalacia presents with inspiratory stridor that typically worsens with feeding, crying, supine positioning, and agitation. The symptoms begin at birth or within the first few weeks of life, peak at 6 to 8 months, and typically resolve by 12 to 24 months [1]. Laryngomalacia is usually diagnosed within the first 4 months of life [2]. Although inspiratory stridor is the classic symptom of laryngomalacia, there are a number of associated symptoms. The most common associated symptoms are related to feeding which include regurgitation, emesis, cough, choking, and slow feedings. Infants with laryngomalacia may have a difficult time coordinating the suck swallow breath sequence needed for feeding as a result of their airway obstruction [3]. The increased metabolic demand of coordinating eating and breathing against the obstruction can be so severe that it results in weight loss and failure to thrive. Other less common but concerning associated symptoms are tachypnea, suprasternal and substernal retractions, cyanosis, pectus excavatum, and obstructive sleep apnea. Chronic hypoxia from airway obstruction can lead to pulmonary hypertension if not recognized and managed.

 It is important for a clinician to differentiate laryngomalacia from other conditions that cause noisy breathing. All too often the diagnosis of tracheomalacia, asthma, bronchiolitis, and reactive airway disease may precede the correct diagnosis of laryngomalacia. Because infants are often misdiagnosed with these conditions, understanding patterns and characteristics of breathing will aid the clinician in differentiating the noisy breathing of laryngomalacia from others. Identifying which phase of the respiratory cycle will also help determine the level of obstruction. Wheezing, stertor, and stridor are the types of noisy breathing. Wheezing is typified as a coarse whistling sound heard on the phase of expiration and is usually due to lung disease. Stertor is a grunting or a snoring sound and is loudest during inspiration. In children it is typically caused by adenotonsillar disease. The high-pitched noise of stridor can occur during the respiratory phase of inspiration, expiration, or both (biphasic). Inspiratory stridor is caused by airway obstruction at the vocal cords or higher. Biphasic stridor is caused by obstruction below the vocal cords. The most common cause of biphasic stridor in children is viral croup. Expiratory stridor is caused by obstruction in the trachea. The most common cause of expiratory stridor in children is tracheomalacia. Infants and children who have chronic stridor should be referred to an otolaryngologist for accurate diagnosis.

## 3. Diagnosis

**Figure 1:**
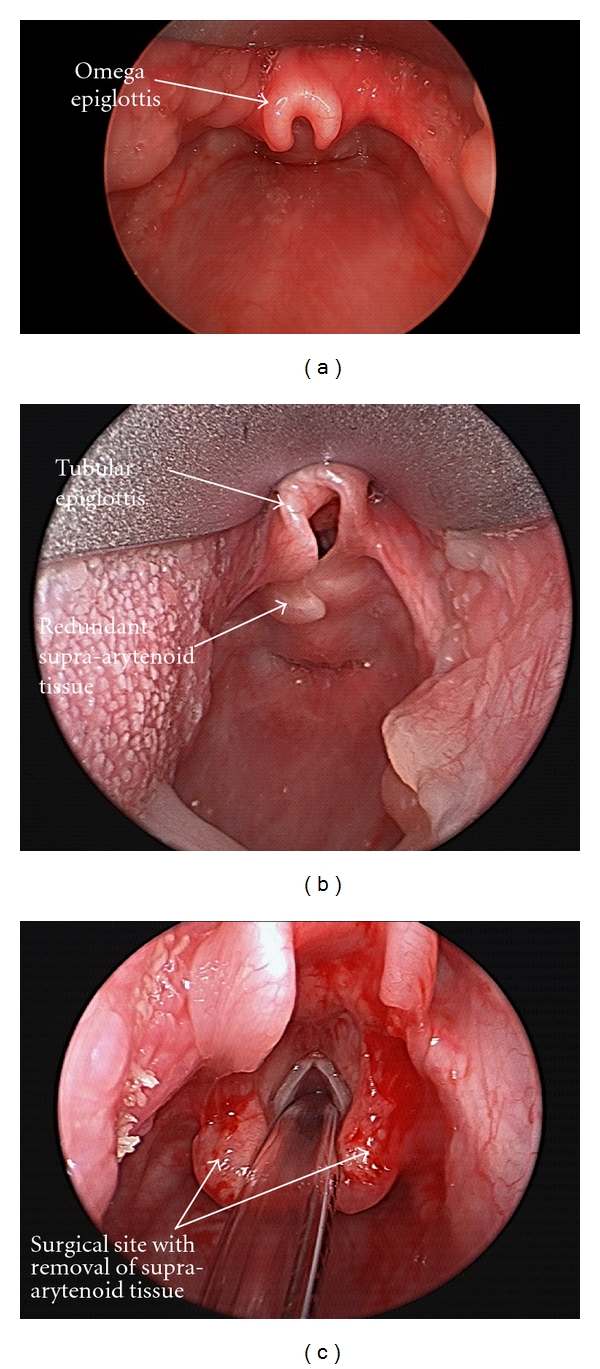
(a) Omega-shaped epiglottis. (b) A tubular-shaped epiglottis along with redundant supra-arytenoid tissue which is obstructing the glottis during inspiration. (c) the site of redundant supra-arytenoid tissue after surgical removal.

The diagnosis of laryngomalacia is suspected by the typical clinical history but is confirmed by flexible laryngoscopy in an awake infant. Flexible laryngoscopy is easily preformed in the otolaryngology office with the help of a caregiver. The infant is held in the caregivers lap in an upright or semireclined position, and a flexible laryngoscope is passed through the nose, pharynx, and positioned above the larynx. The otolaryngologist is able to examine the dynamic movement of the laryngeal structures during spontaneous respiration and differentiate laryngomalacia from other cause of inspiratory stridor such as vocal cord paralysis or a laryngeal cyst. Supraglottic tissue collapse and obstruction during inspiration is the hallmark of laryngomalacia. The epiglottis, false vocal cords, arytenoids, ventricle, and aryepiglottic folds are the structures making up the supraglottis. As seen in Figures 1(a) and 1(b), the common findings seen on exam are prolapse of the posteriorly positioned arytenoid cartilages and mucosa into the airway during inspiration, shortening of the distance between the arytenoid and epiglottis, and an “omega-shaped” or retroflexed epiglottis.

## 4. Etiology

The exact etiology of laryngomalacia is unknown and continues to be an area of great interest and research. Theories of etiology include the anatomic, cartilaginous, and neurologic theories. The anatomic theory proposes that there is an abnormal placement of flaccid tissue resulting in stridor. The challenge with the anatomic theory is there are infants who have the typical anatomic laryngeal findings of laryngomalacia who do not have symptoms of airway obstruction. The cartilaginous theory proposes that the cartilages of the larynx are immature and abnormally pliable. This theory has been refuted by the finding of histologically normal cartilage in infants with symptomatic laryngomalacia. The neurologic theory is the best supported by the literature and as a result is the prevailing etiologic theory [2].

 The neurologic theory recognizes that laryngomalacia may be a consequence of an underdeveloped or abnormally integrated CNS system, particularly the peripheral nerves and brainstem nuclei responsible for breathing and airway patency. As the infant matures laryngomalacia likely resolves secondary to the maturation of the CNS system. The laryngeal adductor reflex is a vagal nerve reflex responsible for laryngeal function and tone. The afferent activation of the reflex is mediated by the superior laryngeal nerve which is located in the aryepiglottic fold [2]. Sensory information from this nerve is then transmitted to the brainstem nuclei that regulate respiration and swallowing. A motor response to sensory stimulation is mediated by the vagus nerve resulting in glottic closure, inhibition of respiration, and swallow. An alteration in this pathway has a role in the etiology of laryngomalacia and the associated feeding symptoms. Laryngeal sensory testing in infants with laryngomalacia has demonstrated that the sensory stimulus threshold needed to elicit the typical motor response is elevated in those with moderate-to-severe disease versus those with mild disease. This testing supports the notion of an underdeveloped or abnormally integrated peripheral and central nervous system mechanism of laryngeal function and tone [2].

## 5. Spectrum of Disease

Laryngomalacia has a disease spectrum that can be divided into mild, moderate, and severe categories [2]. These categories are not based on the quantity of stridor but rather by the associated feeding and obstructive symptoms. Those with mild disease usually have inconsequential inspiratory stridor. Those with moderate disease usually have stridor with feeding-related symptoms and often improve on acid suppression treatment. Those with severe disease require surgical intervention, usually supraglottoplasty. Understanding the spectrum of symptoms and unique patient factors that influence disease severity will help determine which patients may worsen and require referral to an otolaryngologist for further management.

At the time of presentation to a health care provider approximately 40% of infants will have mild laryngomalacia. They present with inspiratory stridor and the occasional feeding-associated symptoms of cough, choking, and regurgitation. They have a coordinated suck swallow breath sequence and feed comfortably. Airway obstruction does not lead to hypoxia. They have an average resting oxygen saturation of 98–100% [4]. Seventy percent of those that present as mild disease will have an uneventful disease course and resolution and can be managed expectantly. The remaining 30% who present with worsening reflux symptoms that interfere with feeding will progress to the moderate disease category. In addition to reflux-related symptoms, those with mild disease and baseline resting SAO_2_ of ≤96% are predicted to progress to the moderate disease category [2, 4].

At the time of presentation up to 40% will have moderate laryngomalacia. Those in this category present with the typical stridor but are described by their caregivers as fussy and hard to feed. They have frequent feeding-associated symptoms of cough, choking, regurgitation, and cyanosis during feeding. If not recognized and managed, feeding problems can lead to aspiration, weight loss, and laborious feedings. Strategies to improve feeding symptoms include pacing, texture modification by thickening formula/breast milk, and upright position for feeding. Acid suppression treatment is effective for which the mechanism is discussed below. Up to 72% percent of infants will have resolution of their symptoms by 12 months utilizing this management strategy. Infants with moderate laryngomalacia are not hypoxic; however they have a lower average resting SAO_2_ of 96% [2, 4]. It is important to carefully monitor this group of infants as up to 28% develop severe disease and have worsening symptoms despite feeding modification and acid suppression therapy. These patients require surgical intervention [3]. An infant with moderate disease and an average resting SAO_2_ of ≤91% is also more likely to require surgical intervention, usually supraglottoplasty [2, 4].

Twenty percent of infants have severe laryngomalacia at the time of presentation to a health care provider. They present with inspiratory stridor and other associated symptoms that include recurrent cyanosis, apneic pauses, feeding difficulty, aspiration, and failure to thrive. Suprasternal and subcostal retractions can lead to pectus excavatum. The average resting baseline SAO_2_ in those with severe disease is 86% [2, 4]. If not recognized and managed, chronic hypoxia can lead to pulmonary hypertension and cor pulmonale. As discussed below those with severe disease will likely require surgical intervention in addition to acid suppression treatment for management. The mainstay for surgical intervention is supraglottoplasty whereby the obstructing collapsing tissue is removed through an endoscope. Tracheotomy is rarely indicated and is reserved for supraglottoplasty failures and those with multiple medical comorbidities [2, 4].

## 6. Medical Comorbidities

In addition to associated symptoms it is important for members of the health care team to recognize that the presence of medical comorbidities impacts symptoms and disease course. Gastroesophageal reflux disease (GERD) and neurologic disease are the most common medical comorbidities. Other comorbidities that influence the outcome are the presence of an additional airway lesion, congenital heart disease, and the presence of a syndrome or genetic disorder.

### 6.1. Gastroesophageal and Laryngopharyngeal Reflux

Gastroesophageal reflux is noted in 65–100% of infants with laryngomalacia [4]. The airway obstruction of laryngomalacia generates negative intrathoracic pressure which promotes gastric acid reflux onto the laryngopharyngeal tissues leading to laryngopharyngeal reflux. The laryngeal tissues are sensitive to the acid exposure and become edematous as a response. Increased supraglottic edema results in further collapsing of these tissues into the airway and further obstructive symptoms. A vicious cycle of increased obstruction, GERD, and edema then ensues. Prolonged acid exposure also blunts laryngeal sensation which decreases the motor response to swallow in response to secretions. Decreased laryngeal sensation explains the coughing and choking during feedings which are commonly seen with laryngomalacia. The vagal reflex responsible for laryngeal tone is also responsible for lower esophageal sphincter tone and esophageal motility [2]. Decreased lower esophageal tone and esophageal dysmotility are known risk factors for GERD and could be a factor in the GERD seen in laryngomalacia patients.

GERD should be treated in all patients with laryngomalacia and feeding symptoms. Upright positioning during feeding and bottles that minimize aerophagia may decrease the number of reflux events. Acid suppression therapy improves symptoms and may shorten the duration of the natural course. There are no controlled studies demonstrating the most effective GERD treatment regimen in laryngomalacia patients. The senior author's experience is to begin infants with feeding symptoms on high-dose histamine type-2 receptor antagonist therapy (ranitidine 3 mg/kg, 3 times a day). A proton pump inhibitor is added for refractory symptoms and breakthrough symptoms. At times a combination of daytime proton pump inhibitor therapy and nighttime histamine type-2 receptor antagonist therapy is used. Most infants are kept on acid suppression therapy for an average of 9 months [4].

In infants with moderate-to-severe disease, complementary gastrointestinal studies may be beneficial in prognosis and management. An esophagram with small bowel follow-through is useful in evaluating reflux and aspiration along with ruling out containment gastrointestinal disorders such as pyloric stenosis. Aspiration during feedings can be evaluated by a videofluoroscopic swallow study or a functional endoscopic swallow study. Aspiration seen on these swallow evaluations may prompt surgical management of the laryngomalacia in order to decrease the respiratory consequences of chronic aspiration into the lung [3]. Twenty-four-hour pH studies and impedance studies may be useful in determining management strategies for the infant with severe reflux despite acid suppression therapy. Impedance testing is a method to detect esophageal bolus movement. When combined with pH studies it is helpful in detecting both acidic and nonacidic gastroesophageal reflux events. Depending on the results of these studies, expanded medical management or fundoplication surgery may be warranted for reflux control.

### 6.2. Neurologic Disease

Neurologic disease is present in 20–45% of infants with laryngomalacia and includes seizure disorder, hypotonia, developmental delay, cerebral palsy, mental retardation, microcephaly, quadriparesis, and Chiari malformation. Neurologic disease may decrease vagal nerve function at the brainstem level contributing to decreased laryngeal tone. Infants with neurologic disease require surgical intervention at higher rates than those without [4]. Neuromuscular hypotonia also leads to collapse of the supporting muscles in the pharynx and swallowing mechanism leading to airway obstruction and feeding symptoms. Those with neurologic disease will often have worse symptoms or a prolonged course of symptoms. Some may not have resolution of their symptoms despite medical intervention or supraglottoplasty. These patients may require accessory routes for feeding and breathing, usually a tracheostomy.

### 6.3. Secondary Airway Lesions

The incidence of secondary or synchronous airway lesions (SAL) in laryngomalacia ranges from 7.5 to 64% [5–9]. The higher range of SAL is likely explained by the technique used for diagnosis and the indication for looking for another lesion. The presence of a SAL can be screened by using airway fluoroscopy for tracheomalacia and high-kilovoltage airway radiographs for fixed structural lesions such as subglottic stenosis. Tracheomalacia is the most common synchronous airway lesion followed by subglottic stenosis. SAL have an accumulative effect on airway obstruction. Airway obstruction from laryngomalacia combined with a SAL can lead to greater airway obstruction with increased negative intrathoracic pressure. Negative intrathoracic pressure potentiates gastroesophageal and laryngopharyngeal reflux. Gastroesophageal and laryngopharyngeal reflux and its complications add to the severity of symptoms previously described [2, 6]. Infants with mild or moderate disease that have a SAL are 4.8 times more likely to require surgical intervention [6]. Diagnosis of SAL may lead to earlier intervention and ultimately affect progression of disease. By surgically addressing laryngomalacia, the resultant effect of SAL on the airway may become less significant. If a SAL is suspected on screening radiographs, the infant will benefit from a referral to an otolaryngologist for clinical correlation.

### 6.4. Congenital Heart Disease

Congenital heart disease is reported in 10% of infants with laryngomalacia. These infants are more likely to have moderate-to-severe disease at the time of presentation. The additive effect of airway obstruction on compromised cardiovascular function likely tips these infants towards worsening symptoms. Up to 34% of infants with both laryngomalacia and congenital heart disease will require surgical management [2].

### 6.5. Congenital Anomalies/Syndromes/Genetic Disorders

Congenital anomalies and genetic disorders occur with an estimated incidence of 8–20% [2, 10, 11]. The incidence is as high as 40% of infants with severe laryngomalacia that require surgical intervention [2, 12]. Infants with congenital anomalies and genetic disorders often have other medical comorbidities such as synchronous airway lesions, cardiac disease, and neurologic disease that confound oxygenation and breathing; this makes any degree of airway obstruction more of problematic for these patients. Infants with severe laryngomalacia, an isolated anomaly or syndrome, and minimal comorbidities can be managed successfully with supraglottoplasty [2, 12]. Of those infants, Down syndrome appears to be the most commonly reported associated genetic disorder with laryngomalacia. Fifty percent of those that have respiratory symptoms also have laryngomalacia [13–15]. The senior author's experience with supraglottoplasty in Down syndrome children is that if no coexisting cardiac disease or neurologic disease is present, they do well with aggressive acid suppression therapy and supraglottoplasty even if a synchronous airway lesion is present. Those with cardiac disease, neurologic disease, and synchronous airway lesions often fail supraglottoplasty and may require a tracheostomy until cardiac disease is treated.

Those with laryngomalacia and syndromes associated with micrognathia such as CHARGE association and Pierre Robin sequence will do worse due to the retrodisplacement of the tongue base. The retrodisplaced tongue base collapses on the epiglottis in addition to supra-arytenoid tissue redundancy and short aryepiglottic folds. Supraglottoplasty or epiglottic suspension procedures usually are unsuccessful [16], and most with severe airway obstruction and laryngomalacia will require a tracheostomy until they grow into the micrognathia or surgical intervention is performed to correct it. Laryngomalacia and variants of 22q11.2 microdeletion syndrome are described to have severe upper airway obstruction [17–19] and can be successfully managed with supraglottoplasty [17]. Because cervical vertebral anomalies are common in this patient population, cervicomedullary compression of the brainstem should be investigated as a potentiating cause of symptoms. A recent case series describes a child who had laryngomalacia symptom reversal and improvement in laryngeal tone after brainstem decompression and did not require supraglottoplasty [19].

If micrognathia is not present, a syndrome or anomaly should not preclude supraglottoplasty in those with severe laryngomalacia that require intervention. The rates of failure and tracheostomy placement however may be higher in these patients and should be taken into consideration when counseling parents and managing this unique group of infants.

## 7. Surgical Management

Surgical management is indicated in those with severe disease. The most common indications for surgery are stridor with respiratory compromise and feeding difficulties with failure to thrive [1]. Severe airway obstruction with significant retractions, pectus excavatum, cor pulmonale, pulmonary hypertension, and hypoxia are all considered absolute indications for surgery. The relative indications are aspiration with recurrent pneumonia, weight loss without true failure to thrive, and a difficult to feed child who has not responded to acid suppression therapy. The decision to operate is individualized and based on the trend of the infants overall health and development. Supraglottoplasty is the mainstay of surgical treatment for laryngomalacia. The patient is anesthetized with a combination of mask and intravenous anesthesia. The airway is first evaluated by rigid endoscopy (microdirect laryngoscopy and bronchoscopy) to rule out secondary lesions of the subglottis and trachea. The supraglottis is visualized during spontaneous respiration, and the major areas of collapse are noted. The larynx is then exposed with operating laryngoscopes, and the supraglottoplasty is performed focusing on removal of the redundant arytenoid mucosa. As seen in Figure 1(c), the procedure is tailored to the patient's areas of obstruction, and care is taken to preserve mucosa in areas prone to stenosis. The success of supraglottoplasty approximates 94% and has a low complication rate [1]. Revision supraglottoplasty or tracheostomy will be required in 19–45% of infants and is directly influenced by the number and type of medical comorbidities [2]. Tracheostomy is reserved for patients who continue to have life-threatening airway obstruction and who fail to improve after supraglottoplasty.

## 8. Conclusion

Laryngomalacia is a common disease of infancy where the diagnosis is suspected by primary care providers based on history. Those with mild disease can be managed expectantly. Continued monitoring of the symptoms is necessary as symptoms can progress over the natural course of the disease. Recognizing patient factors and symptoms associated with moderate and severe disease helps determine which infants will benefit from otolaryngology consultation. Identifying patient factors that influence disease severity and outcomes is an important aspect of counseling care givers and providing care to infants with laryngomalacia.
